# A Systematic Approach to the Evaluation of Acute Musculoskeletal Injuries in the Emergency Department

**DOI:** 10.7759/cureus.111769

**Published:** 2026-06-29

**Authors:** Daniel Rossie

**Affiliations:** 1 Emergency Medicine, Loma Linda University Medical Center, Loma Linda, USA

**Keywords:** achilles tendon rupture, emergency sports medicine, musculoskeletal injuries, ottawa ankle rules, point-of-care ultrasound (pocus)

## Abstract

Musculoskeletal (MSK) complaints account for a significant proportion of emergency department (ED) visits and range from minor soft tissue injuries to fractures, tendon ruptures, dislocations, septic arthritis, and compartment syndrome. Despite their frequency, these presentations can be diagnostically challenging because of variable mechanisms of injury, overlapping examination findings, and limitations of plain radiography for detecting soft tissue and occult bony injuries. Missed diagnoses, such as Achilles tendon rupture, scaphoid fracture, Lisfranc injury, and tibial plateau fracture, may result in substantial morbidity, while excessive imaging contributes to unnecessary cost and resource utilization. This review presents a practical framework for evaluating acute MSK injuries in the ED. The approach emphasizes five core components: mechanism of injury, functional assessment, focused physical examination, targeted imaging, and disposition planning. Validated clinical decision tools, including the Ottawa Ankle and Knee Rules, are reviewed, along with the evolving role of point-of-care ultrasound in emergency MSK evaluation. High-risk injuries that are commonly missed in the ED are discussed in detail, with emphasis on recognition, imaging strategy, and early management. By applying a structured diagnostic approach, emergency physicians can improve diagnostic accuracy, reduce unnecessary imaging, and better identify injuries requiring urgent intervention or specialty follow-up.

## Introduction and background

Musculoskeletal (MSK) complaints represent a substantial proportion of emergency department (ED) visits and encompass a wide range of injuries. Estimates suggest that MSK injuries account for approximately 20-25% of ED presentations, making them one of the most common categories of emergency care [1]. Despite their frequency, evaluation of these injuries can be challenging due to variability in mechanisms of injury, subtle examination findings, and limitations of early imaging.

Emergency physicians must rapidly identify injuries that require urgent intervention while avoiding unnecessary imaging and specialty consultation. Missed or delayed diagnosis of significant MSK injuries, including tendon ruptures, occult fractures, and ligamentous instability, can lead to long-term morbidity and functional impairment. At the same time, excessive imaging contributes to healthcare costs and ED crowding.

Traditional approaches to MSK injury evaluation often focus on individual body regions. While this method may be useful in subspecialty practice, it can be inefficient in the ED, where clinicians must evaluate injuries across multiple anatomic regions within time constraints. A structured diagnostic framework based on the mechanism of injury, functional assessment, focused physical examination, and judicious imaging can improve efficiency and diagnostic accuracy [2].

**Figure 1:**
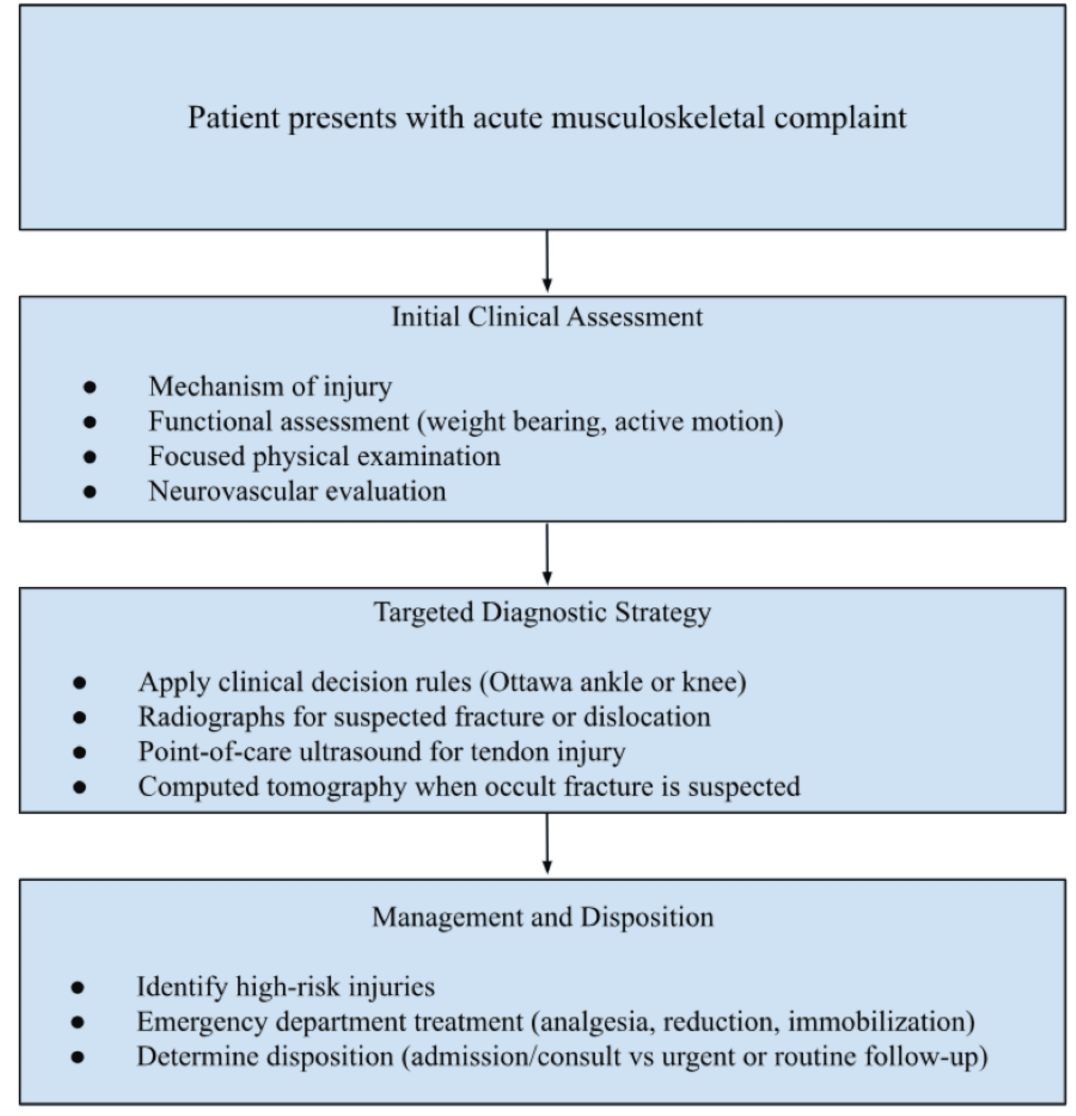
Structured framework for the evaluation of acute musculoskeletal injuries in the emergency department, integrating mechanism of injury, functional assessment, focused physical examination, and targeted imaging to guide management and disposition. Created with Google Sheets (Google, Inc., Mountain View, CA)

This review presents a practical approach to the evaluation of both traumatic and non-traumatic, acute MSK pathology in the ED. The goal is to provide emergency physicians with a method for rapidly identifying clinically significant injuries while guiding appropriate imaging and disposition (Figure 1).

## Review

Mechanism of injury

Understanding the mechanism of injury is a critical first step in the evaluation of MSK trauma. Mechanism not only narrows the differential diagnosis before physical examination begins, but also helps identify injuries that may not be immediately apparent on initial assessment or plain radiography. Emergency physicians should obtain a focused injury history, including the direction and magnitude of force, extremity position at the time of injury, immediate symptoms, and functional ability afterward.

Direct Trauma

Direct trauma occurs when an external force impacts a specific anatomic structure. These injuries are commonly encountered in contact sports, motor vehicle collisions, falls, and workplace incidents. The severity of injury is broadly proportional to the energy of the applied force, though host factors such as bone density, patient age, and soft tissue coverage also modulate injury patterns.

Typical injuries include fractures, contusions, lacerations, and ligamentous disruptions at the site of impact. Localized tenderness corresponding precisely to the point of contact is a useful clinical finding and, when overlying a bone, warrants careful consideration of fracture. Emergency physicians should assess not only the primary injury site but also the joint proximal and distal to the impact, as transmitted forces can cause injury remote from the initial point of contact.

Rotational or Twisting Injuries

Rotational forces commonly occur during pivoting, rapid direction changes, or sudden deceleration in sports such as basketball, soccer, football, and skiing. These mechanisms place torsional stress on ligaments, menisci, tendons, and the joint capsule.

Common injuries include anterior cruciate ligament (ACL) tears, meniscal injuries, lateral ankle sprains, and syndesmotic injuries. Patients frequently describe a twisting motion associated with immediate pain, instability, or a popping sensation. The direction of force and location of pain can help localize the injured structure and guide examination.

Eccentric Muscle Loading

Eccentric loading occurs when a muscle contracts while lengthening under tension. This mechanism places substantial stress on the musculotendinous unit and is commonly responsible for tendon rupture and significant muscle injury.

Classic examples include Achilles tendon rupture during sudden push-off, quadriceps or patellar tendon rupture during forced flexion against resistance, and proximal hamstring injury during sprinting. These injuries often occur in recreational athletes and may initially appear deceptively benign because external signs of trauma are limited.

Axial Loading

Axial loading injuries result from compressive forces transmitted along the longitudinal axis of a bone or joint. These mechanisms are commonly associated with falls from height, jumping injuries, and high-energy trauma.

Important injuries include tibial plateau fractures, calcaneal fractures, Lisfranc injuries, and vertebral compression fractures. Patients may have relatively subtle examination findings despite clinically significant injury, particularly when articular surfaces are involved. Persistent pain or inability to bear weight after axial loading should prompt careful evaluation even when initial radiographs appear normal.

Hyperextension and Avulsion Mechanisms

Hyperextension injuries occur when a joint is forced beyond its normal range of motion, placing stress on ligaments, tendons, capsules, and adjacent neurovascular structures. These mechanisms are seen in posterior knee injuries, elbow trauma, cervical spine trauma, and some finger injuries. In the cervical spine, hyperextension can produce significant ligamentous or cord injury despite minimal findings on plain radiography, making clinical decision tools and appropriate imaging important.

Avulsion injuries occur when a tendon or ligament pulls away a fragment of bone at its attachment site. These injuries are especially common in pediatric patients because the physis is often weaker than the surrounding ligaments. In adults, avulsion patterns can still provide important diagnostic clues, such as a Segond fracture suggesting ACL injury or a fifth metatarsal base avulsion from peroneus brevis traction. Recognizing these patterns on plain radiographs can help identify the associated soft tissue injury.

Functional assessment

After identifying the mechanism of injury, the next critical step in MSK evaluation is assessment of functional deficits. Functional assessment often provides early clues to the severity of injury and may help differentiate minor soft tissue injuries from more significant ligamentous, tendon, or bony pathology. Functional deficits may provide more clinically useful information than pain intensity alone, particularly when interpreted together with the mechanism of injury and physical examination. 

Weight-Bearing Ability

For lower extremity injuries, the patient's ability to bear weight is among the most diagnostically useful early questions. Inability to bear weight following acute trauma raises concern for fracture, complete ligamentous disruption, or tendon rupture, and is a key component of the Ottawa Ankle and Knee Rules. Weight-bearing assessment should be performed deliberately: the clinician should ask whether the patient was able to take steps at the time of injury and whether they are able to bear weight at the time of examination. These are distinct time points, and both are incorporated into validated decision tools.

It is important to recognize that weight-bearing assessment has limitations. Patients with low pain tolerance may refuse weight-bearing in the setting of minor injuries, while athletes or stoic individuals may briefly weight-bear despite a significant fracture. The finding should therefore be interpreted in context with the remainder of the examination rather than in isolation.

Active Range of Motion

Assessment of active range of motion identifies deficits in the volitional motor function of joints and the integrity of the musculotendinous units responsible for movement. Patients with tendon ruptures often retain passive motion of the affected joint but are unable to actively move the joint against gravity, a dissociation that is highly characteristic of complete disruption. Examples include the inability to actively extend the knee in patellar tendon rupture, loss of active dorsiflexion in peroneal tendon subluxation, and the apparent preservation of weak plantarflexion in Achilles tendon rupture through accessory muscles.

In contrast, significant limitations of both active and passive range of motion suggest joint effusion, periarticular fracture, or dislocation. Fractures with intra-articular extension are particularly likely to produce guarded, restricted motion in all planes. Evaluating the range of motion early in the examination also allows the clinician to determine which specific movements reproduce or exacerbate pain, directing subsequent examination maneuvers.

Neurovascular Status

Baseline neurovascular assessment should be performed for all significant extremity injuries, particularly those involving high-energy mechanisms, fractures, and dislocations. Examination should include evaluation of distal pulses, capillary refill, skin color and temperature, and light touch sensation in the distal distribution of major peripheral nerves. Motor testing of distal muscle groups should also be performed when the mechanism raises concern for nerve injury.

Neurovascular compromise at initial presentation represents an emergency. Vascular injury can accompany knee dislocations (popliteal artery), posterior elbow dislocations, and supracondylar humerus fractures in children. Nerve injuries are associated with glenohumeral dislocations (axillary nerve), fibular head fractures (common peroneal nerve), and posterior hip dislocations (sciatic nerve). Documenting baseline neurovascular status before any reduction or manipulation is both clinically important and medicolegally essential.

Stability and Gait

A subjective sensation of instability following injury, particularly with twisting mechanisms at the knee or ankle, suggests ligamentous disruption. Patients with significant ACL injury often describe the knee "giving way" during pivoting or deceleration, while those with a lateral ankle ligament tear may report the ankle turning under them repeatedly. Observations of gait, when safely obtainable, can reveal antalgic patterns, inability to push off, or abnormal joint mechanics that support the clinical assessment.

Physical examination considerations

The physical examination remains the cornerstone of MSK injury evaluation in the ED. Although imaging is often necessary, many clinically significant injuries can be suspected through a structured examination. A focused exam should assess inspection, palpation, range of motion, strength, neurovascular status, and functional integrity of the affected extremity. Examination findings should always be interpreted within the context of the injury mechanism and the patient's functional limitations (Table 1). Note: The diagnostic performance of these maneuvers may vary depending on the acute phase of injury, pain, swelling, guarding, patient cooperation, and examiner experience. This is particularly relevant for ligamentous tests of the knee, ankle, and shoulder. 

**Table 1:** High-yield physical examination maneuvers for musculoskeletal injuries in the emergency department. ACL: anterior cruciate ligament, AC joint: acromioclavicular joint, TMT: tarsometatarsal

Injury Suspected	Examination Maneuver	Key Finding
ACL tear	Lachman test	Increased anterior tibial translation at 20° flexion
Meniscal injury	McMurray test	Pain or click with rotation and flexion
Achilles tendon rupture	Thompson test	Absence of plantarflexion with calf squeeze
Patellar tendon rupture	Active extension assessment	Inability to extend knee against gravity
AC joint injury	Cross-body adduction test	Reproduced pain at AC joint
Shoulder instability	Apprehension test	Apprehension with external rotation in abduction
Scaphoid fracture	Snuffbox palpation; axial thumb loading	Localized tenderness; pain with thumb compression
Syndesmotic injury	External rotation stress test; squeeze test	Pain above ankle joint line
Lisfranc injury	Piano key test; midfoot stress	Pain with dorsal-plantar stress at TMT joints
Medial collateral ligament	Valgus stress test	Medial joint line gapping or pain
Posterior cruciate ligament	Posterior drawer test	Increased posterior tibial translation

Inspection

Inspection begins the moment the patient is encountered. Emergency physicians should assess the overall positioning of the extremity, the willingness to move the injured area, visible deformity, swelling, ecchymosis, skin integrity, and asymmetry compared with the contralateral side. Gross deformity may indicate fracture or dislocation, while more subtle findings, such as joint effusion or localized swelling, can help narrow the differential diagnosis.

Skin examination is particularly important in trauma. Abrasions, lacerations, skin tenting, or open wounds near fractures may indicate open injury requiring urgent management. Ecchymosis patterns may also provide diagnostic clues, such as plantar ecchymosis in Lisfranc injury or distal migration of bruising in muscle or tendon rupture.

Palpation

Palpation is used to identify focal areas of tenderness and to differentiate bony from soft tissue injury. Systematic palpation of the injured region should include evaluation of adjacent joints and surrounding structures. Point tenderness over a bone should raise suspicion for fracture, particularly in the setting of trauma. In contrast, diffuse tenderness along muscle or ligament structures may suggest strain or sprain. Palpation may also reveal joint effusion, crepitus, or step-off deformities associated with fractures or ligamentous injury. For certain injuries, specific palpation findings may be diagnostic. For example, tenderness in the anatomic snuffbox following wrist trauma raises concern for scaphoid fracture, even when initial radiographs appear normal.

Range of Motion

Both active and passive range of motion should be assessed when tolerated. Inability to actively move a joint may indicate fracture, dislocation, tendon rupture, or severe pain inhibition. Preserved passive range of motion with loss of active motion may suggest disruption of the extensor mechanism or tendon injury.

Pain with passive stretch is an important finding in compartment syndrome and should not be overlooked in patients with significant swelling or high-risk mechanisms. Mechanical blocks to motion may indicate intra-articular loose bodies, locked meniscal injury, or fracture-dislocation.

Strength and Functional Testing

Strength testing provides additional information regarding tendon, muscle, and neurologic integrity. Functional testing is often more useful than isolated strength grading in the acute setting. The ability to perform a straight leg raise, actively extend the knee, plantarflex the ankle, or bear weight may help identify clinically significant injuries even when imaging findings are subtle.

Weight-bearing status is particularly important in lower-extremity injuries. Inability to bear weight after ankle, foot, hip, or knee trauma should increase suspicion for fracture or instability, even when radiographs are initially normal.

Neurovascular Examination

A complete neurovascular examination is essential in all MSK injuries. Distal pulses, capillary refill, motor function, and sensation should be documented carefully before and after reduction or splinting procedures.

Certain injuries carry predictable neurovascular complications. Knee dislocations may injure the popliteal artery, supracondylar humerus fractures may involve the brachial artery or anterior interosseous nerve, and shoulder dislocations may affect the axillary nerve. Neurologic deficits may initially be subtle and can evolve over time, making reassessment important in high-risk injuries.

Regional Examination

A structured regional examination improves diagnostic consistency and reduces the likelihood of missed injuries. Examination should progress systematically through the affected joint and the structures above and below the injury site.

Shoulder: Shoulder examination begins with inspection for asymmetry, deformity, or guarding. Active and passive range of motion should be assessed when tolerated. Patients with rotator cuff injury often demonstrate pain or weakness with resisted abduction or external rotation, while instability may be reproduced with apprehension testing. Posterior shoulder dislocation should be considered after seizure, electrocution, or trauma when the arm is held in internal rotation, and the patient cannot externally rotate.

Elbow and forearm: Elbow examination should assess the lateral epicondyle, medial epicondyle, radial head, and olecranon-the three bony landmarks forming the "elbow triangle." Disruption of this equilateral triangle relationship in extension indicates dislocation or fracture-dislocation. Radial head fractures, which are common following a fall onto an outstretched hand (FOOSH), may present with only lateral elbow pain and limited forearm rotation. The anterior fat pad sign or posterior fat pad sign on a lateral elbow radiograph suggests occult intra-articular injury. Forearm injuries should prompt evaluation of both the wrist and elbow, as Monteggia (proximal ulna fracture with radial head dislocation) and Galeazzi (radial shaft fracture with distal radioulnar joint disruption) fracture-dislocations require recognition of the joint injury rather than the fracture alone.

Wrist and hand: Evaluation of the wrist and hand should include inspection for swelling, deformity, rotational abnormalities, and soft tissue injury. Snuffbox tenderness, scaphoid tubercle tenderness, or pain with axial thumb loading should raise suspicion for scaphoid fracture even with normal radiographs. Tendon function, finger cascade, capillary refill, and sensory examination should be assessed routinely.

Hip and pelvis: Hip pathology should be considered in patients with groin pain, inability to bear weight, or pain with axial loading of the femur. Occult femoral neck fractures may present with relatively subtle findings despite normal radiographs. Marked pain with passive hip motion (log rolling the affected side) or inability to ambulate should prompt consideration of advanced imaging when suspicion remains high.

Knee: Knee examination should assess the extensor mechanism integrity (patellar and quadriceps tendons), medial and lateral collateral ligaments, anterior and posterior cruciate ligaments, and the medial and lateral joint lines (menisci). A rapidly developing effusion or hemarthrosis should raise concern for ACL injury, osteochondral fracture, patellar dislocation, or intra-articular fracture. The Lachman test, performed at 20-30 degrees of flexion, is the most sensitive manual test for ACL injury and is more reliable than the anterior drawer test in the acutely injured, guarded knee.

Ankle and foot: Ankle examination should include palpation of the posterior malleoli, base of the fifth metatarsal, navicular, syndesmosis, and midfoot structures. Ligamentous testing may help identify lateral ankle instability or syndesmotic injury. Midfoot tenderness, plantar ecchymosis, or pain with forefoot stress should raise concern for Lisfranc injury even when initial radiographs are normal.

Clinical Application of Examination Findings in the ED

No single examination maneuver is perfectly sensitive or specific. Examination findings should be integrated with the mechanism of injury, functional assessment, and imaging results. A structured physical examination allows emergency physicians to identify high-risk injuries more efficiently, improve diagnostic accuracy, and guide appropriate imaging, treatment, and disposition decisions.

Imaging strategy in the ED

Imaging is an important adjunct to focused history and physical examination in acute MSK injury, but it should complement rather than replace clinical assessment. The core principle is that imaging should be obtained when it will meaningfully influence diagnosis, management, or disposition - not as a default response to pain or patient expectation. Thoughtful application of validated clinical decision tools, awareness of the limitations of specific modalities, and appropriate escalation to advanced imaging when clinical suspicion persists despite normal plain films define the standard of care (Table 2).

**Table 2:** Imaging guidance for common musculoskeletal injuries in the emergency department. FOOSH: fallen onto an outstretched hand, POCUS: point-of-care ultrasound, AP: anteroposterior, MRI: magnetic resonance imaging, CT: computed tomography, NEXUS: National Emergency X-Radiography Utilization Study

Injury/Region	First-Line Modality	Notes and Escalation
Ankle trauma	Plain radiograph	Apply Ottawa Ankle Rules; three views standard
Knee trauma	Plain radiograph	Apply Ottawa Knee Rules; MRI deferred to outpatient
FOOSH wrist injury	Plain radiograph (three views)	Immobilize and treat as a scaphoid fracture if there is snuffbox tenderness with a normal radiograph
Suspected Achilles rupture	Clinical exam ± POCUS	POCUS if diagnosis equivocal; MRI deferred to outpatient
Midfoot/Lisfranc injury	Weight-bearing AP foot (if tolerated), along with multiple views looking for other injuries	Can consider contralateral radiograph for comparison; CT if plain film equivocal and suspicion remains
Tibial plateau fracture	Plain radiograph	Low threshold for CT if plain films is inconclusive
Elderly hip injury (negative radiograph)	MRI (preferred) or CT	MRI has the highest sensitivity for occult femoral neck fracture
Cervical spine trauma	CT (preferred over plain films)	Apply the NEXUS or Canadian Cervical Spine Rule to guide imaging
Elbow injury	Plain radiograph (three views)	Posterior fat pad sign on the lateral view suggests an occult fracture

Plain Radiography

Plain radiography remains the first-line imaging modality for suspected fracture or dislocation due to its wide availability, low cost, and rapid acquisition. Radiographs are generally indicated when there is focal bony tenderness, deformity, inability to bear weight, significant periarticular swelling, reduced range of motion out of proportion to the presumed soft tissue injury, or a mechanism suggesting axial load, high-energy trauma, or direct skeletal impact.

Most joint injuries should be imaged with a minimum of two orthogonal views. Three views (anteroposterior (AP), lateral, and oblique) are standard for the ankle, wrist, and foot. For suspected dislocations, post-reduction films are mandatory to confirm joint congruency and to identify associated fractures not visible on pre-reduction imaging. Comparative views of the contralateral extremity are rarely necessary in adults but may be helpful in pediatric patients when normal physeal anatomy is uncertain.

Point-of-Care Ultrasound (POCUS)

POCUS has an expanding role in emergency MSK evaluation, particularly for suspected tendon injury. Its major advantages are immediate bedside availability, absence of radiation, dynamic real-time imaging capability, and the ability to directly visualize soft tissue structures not assessable by plain radiography. In centers where emergency physician POCUS training is available, it represents a valuable complement to clinical examination.

Achilles tendon rupture is the most established application of POCUS in MSK emergency medicine. High-frequency linear probe imaging of the posterior ankle in longitudinal and transverse planes allows direct visualization of the tendon; a complete rupture appears as a hypoechoic gap within the tendon substance, with the frayed ends visible in real time. Studies have reported sensitivity and specificity both exceeding 90% for complete rupture; however, this may be influenced by operator experience and local training standards [3]. POCUS is particularly useful when the Thompson test is equivocal or the tendon defect is not palpable.

Additional useful POCUS applications in the MSK setting include detection of joint effusion (particularly the knee and hip), identification of large soft tissue hematomas, assessment of extensor mechanism integrity, guidance for arthrocentesis, and detection of posterior shoulder dislocation when standard views are inconclusive. POCUS does not replace dedicated MSK ultrasound for complex soft tissue assessment but provides rapid, actionable information at the bedside.

Computed Tomography (CT)

CT should be reserved for situations in which plain radiographs are negative or indeterminate but clinical suspicion for a significant bony injury remains high. In practice, this most commonly applies to suspected tibial plateau injury, occult calcaneal fracture, complex periarticular fractures with suspected intra-articular extension, subtle Lisfranc disruption, and acute cervical spine injury evaluation when plain film findings are indeterminate or inadequate. CT provides superior resolution of bony architecture, allows multiplanar reconstruction, and can identify fracture lines not visible on two-dimensional plain film.

For patients with significant trauma mechanisms and suspected occult fractures that have not been excluded by plain radiography and clinical reassessment, CT is the appropriate next step before discharge. The radiation exposure, while not negligible, is justified when the clinical consequence of a missed fracture is substantial.

Magnetic Resonance Imaging (MRI)

MRI is rarely required during the initial ED visit for isolated extremity injuries. The indications for emergent or urgent MRI from the ED are limited but include suspected spinal cord compression or nerve root compromise, concern for infectious arthritis with equivocal arthrocentesis, and suspected cauda equina syndrome. For most soft tissue injuries, including ACL tears, meniscal pathology, and partial tendon tears, MRI is the gold standard imaging modality, but it is appropriately deferred to the outpatient setting after specialist evaluation [4].

One exception worth noting is the suspected occult hip fracture in an elderly patient. When plain radiographs and CT are negative and clinical suspicion remains high (inability to bear weight, groin or thigh pain, restricted internal rotation), MRI is highly sensitive for occult femoral neck and intertrochanteric fractures and should be considered before discharge to reduce the risk of missed fracture and delayed diagnosis [4].

Clinical Decision Rules

The Ottawa Ankle Rules remain the best-validated and most widely used clinical decision tool in MSK emergency medicine. Ankle radiographs are indicated when there is pain in the malleolar zone plus either tenderness at the posterior edge or tip of either malleolus, or inability to bear weight for four steps, both immediately after injury and at the time of examination. Foot radiographs are indicated when there is pain in the midfoot zone plus tenderness at the base of the fifth metatarsal or the navicular, or inability to bear weight as above. These rules have demonstrated near-100% sensitivity for clinically significant fractures while reducing unnecessary radiography by approximately 30-40% when applied correctly [5,6]. They should not be applied to patients with diminished sensation or patients with distracting injuries. In children, these rules should be applied only in age groups and clinical settings in which they have been validated, and with caution when cooperation, communication, or examination reliability is limited.

The Ottawa Knee Rules are also a useful clinical decision tool in the ED. Knee radiographs are recommended in patients with acute knee injury if any of the following are present: age 55 years or older, isolated patellar tenderness, tenderness at the fibular head, inability to flex the knee to 90 degrees, or inability to bear weight for four steps at the time of examination. These rules were designed to exclude fracture and do not address the assessment of ligamentous or meniscal injury [7]. Their specificity is lower than the Ottawa Ankle Rules, but their high sensitivity makes them useful for safely reducing unnecessary knee radiography.

High-risk injuries not to miss

Although many MSK injuries presenting to the ED are minor, several clinically important diagnoses are missed with enough frequency to warrant deliberate attention during every evaluation. These injuries are often overlooked because the mechanism seems minor, initial radiographs are negative, or the patient retains some preserved motion or ability to bear weight. The goal of the emergency physician is not merely to label the injury, but to recognize patterns that require immobilization, urgent follow-up, or specialist consultation.

Achilles Tendon Rupture

Achilles tendon rupture is among the most commonly missed diagnoses in emergency MSK care. The incidence has increased over recent decades and is estimated at 6-18 per 100,000 population annually, with the highest rates in middle-aged men engaged in intermittent recreational sport. The typical patient is a 30-50-year-old male participating in a stop-and-start activity, such as basketball, tennis, or racquetball, though ruptures also occur during activities as low-energy as stepping off a curb [8].

The injury occurs most commonly 2-6 cm proximal to the calcaneal insertion, in the area of relative avascularity known as the watershed zone. Risk factors include fluoroquinolone use, corticosteroid injection, inflammatory arthropathy, and prior tendinopathy. The classic mechanism is sudden eccentric loading during push-off or deceleration, producing an abrupt, severe posterior ankle pain often described as feeling like being kicked or struck from behind [8].

The diagnosis is frequently missed in the ED because patients can often weakly plantarflex through accessory muscles (flexor hallucis longus, peroneal tendons), leading to the incorrect conclusion that tendon continuity is preserved. Reports suggest that up to 25% of Achilles ruptures are missed at initial presentation [8]. The examination must include the Thompson test: with the patient prone and the knee flexed to 90 degrees, squeezing the calf muscle should produce plantarflexion of the foot. A positive result (absent plantarflexion) is highly specific for complete rupture, but even a clearly positive Thompson test is sometimes disregarded when patients retain some active foot motion.

While there are exam findings that can increase the pretest probability, they are not sensitive enough to use in isolation. When clinical uncertainty exists, bedside ultrasound has excellent sensitivity and specificity for complete rupture and should be performed at the bedside.

Emergency management includes immobilization in plantarflexion (typically in an equinus boot or posterior splint), restriction of weight-bearing, and urgent orthopedic or sports medicine follow-up within 48-72 hours. Definitive treatment may be surgical or conservative, depending on patient factors, but the choice should be made in conjunction with the specialist. Mislabeling these injuries as ankle sprains can delay definitive treatment and worsen outcomes.

Scaphoid Fracture

Scaphoid fractures are the most common carpal fractures and are frequently complicated by normal initial radiographs at the time of presentation [9]. They occur predominantly in young adults and are typically the result of FOOSH with the wrist in extension and radial deviation. Despite their frequency, scaphoid fractures are notorious for initial radiographic occult presentation: studies estimate that standard wrist radiographs fail to identify 10-20% of scaphoid fractures at the time of initial presentation [10].

The anatomic reason for this difficulty is the complex three-dimensional shape of the scaphoid, which makes complete visualization on standard two-dimensional radiographs challenging. When plain films are obtained, the scaphoid fat pad sign (obliteration of the radiolucent line adjacent to the scaphoid) may be the only radiographic clue, and it is neither sensitive nor specific. Dedicated scaphoid views (posteroanterior with ulnar deviation) increase visibility but do not eliminate false-negative rates.

The clinical consequences of a missed scaphoid fracture are serious. The proximal pole of the scaphoid relies on distal-to-proximal retrograde blood supply, and fractures that disrupt this supply may result in avascular necrosis of the proximal fragment [11]. Delayed union or nonunion, often with progressive carpal collapse (scapholunate advanced collapse (SLAC)), is the natural history of an untreated fracture. Long-term disability and chronic wrist pain are common sequelae.

The practical ED approach is straightforward: patients with a FOOSH mechanism, anatomic snuffbox tenderness, scaphoid tubercle tenderness, or pain with axial thumb loading should be treated as having a scaphoid fracture until proven otherwise. This means thumb spica splinting or commercial scaphoid brace, non-weight-bearing (NWB) on the upper extremity, patient counseling that the initial X-ray may appear falsely negative, and explicit follow-up within 10-14 days for repeat imaging or MRI. When clinical urgency or resource availability allows, MRI is the gold standard for early definitive exclusion of scaphoid fracture and may be arranged from the ED when available.

Tibial Plateau Fracture

Tibial plateau fractures commonly result from axial loading mechanisms and may be associated with significant occult injury despite relatively subtle radiographic findings [12]. They result from axial load, frequently combined with a valgus or varus force, and are seen in motor vehicle collisions, falls from height, and sports injuries. The classic mechanism of a valgus force applied to the knee while bearing weight (the "bumper fracture") produces a lateral plateau injury, the most common type. Medial plateau fractures require higher energy and are associated with more significant ligamentous and neurovascular injury.

Tibial plateau fractures are clinically significant for two reasons beyond the fracture itself. First, they are commonly associated with ligamentous injuries of the knee, including ACL and collateral ligament tears, and with meniscal disruption, occurring in up to 50% of cases. Second, the popliteal neurovascular structures are at risk, particularly with high-energy lateral and bicondylar fractures. Plain radiographs may fail to demonstrate nondisplaced fractures, subtle depression of the articular surface, or fracture lines at the tibial margin. The lateral plateau depression sign and lipohemarthrosis on a cross-table lateral view can be subtle. The emergency physician should maintain a low threshold for CT when the mechanism is consistent with axial load, there is a large knee effusion, the patient cannot bear weight, or plain radiographs are indeterminate. CT with multiplanar reconstruction provides definitive characterization of fracture pattern and articular depression, which guides surgical planning. Initial ED management consists of knee immobilization (posterior splint or knee immobilizer), strict NWB, analgesia, and urgent orthopedic referral [12].

Lisfranc Injury

The Lisfranc joint complex stabilizes the tarsometatarsal joints, particularly the articulation between the medial cuneiform and the base of the second metatarsal. Disruption of this complex can produce instability of the midfoot arch and may occur with either bony injury or purely ligamentous disruption.

Lisfranc injuries are significant because they are frequently missed at initial ED evaluation and because the consequences of delayed or missed diagnosis are severe. The incidence is estimated at one per 55,000 per year, though this likely represents an underestimate given the frequency of missed diagnoses [10]. Mechanisms include direct axial load on a plantar-flexed foot, such as when a foot is trapped in a stirrup during a fall from a horse, a twisting injury during sports, or a crush mechanism. Low-energy twisting mechanisms in athletic activity may produce purely ligamentous injuries with no bony avulsion.

The most characteristic clinical finding is plantar ecchymosis at the arch of the foot, which is highly specific for Lisfranc injury when present, though it may not appear for 24-48 hours [13]. Tarsometatarsal joint line tenderness, pain with piano key stress testing (dorsal-plantar pressure on individual metatarsal heads), and inability to bear weight out of proportion to a presumed sprain are additional key findings. Standard NWB radiographs may appear normal; weight-bearing AP views of the foot are the standard diagnostic study when the patient can tolerate them, and a diastasis greater than 2 mm between the first and second metatarsal bases, or any step-off at the tarsometatarsal joint, is diagnostic. CT is the preferred modality when weight-bearing radiographs are inconclusive or non-obtainable.

ED management requires immobilization in a posterior splint, strict NWB on the affected extremity, and urgent (within 48-72 hours) orthopedic follow-up. Purely ligamentous Lisfranc injuries without fracture require surgical stabilization in most cases to prevent chronic instability and arthritis. Bony Lisfranc injuries with displacement are treated surgically. Dismissing midfoot pain as a simple sprain - particularly in the presence of plantar ecchymosis or inability to bear weight-is a critical diagnostic error.

Septic Arthritis 

Septic arthritis is a joint space infection requiring prompt recognition, aspiration, and antibiotic therapy, often with surgical irrigation and debridement. It is the most important non-traumatic MSK emergency presentation. The annual incidence is approximately 6-7 per 100,000 in the general population, with higher rates in immunocompromised individuals, intravenous drug users, patients with rheumatoid arthritis, and those with prior joint arthroplasty [14]. The knee is the most commonly affected joint (approximately 50% of cases), followed by the hip, ankle, and wrist.

The classic presentation includes an acutely painful, erythematous, warm, and swollen joint with severely restricted and exquisitely painful range of motion. However, fever is absent in a substantial proportion of cases, and systemic toxicity may be mild or absent early in the course. The Kocher criteria were developed for pediatric hip pain and should not be directly generalized to adults, but the underlying features of fever, inability to bear weight, elevated inflammatory markers, and leukocytosis remain clinically relevant when estimating pretest probability.

The differential diagnosis includes crystal arthropathy (gout, pseudogout), reactive arthritis, and traumatic hemarthrosis. Crystal arthropathy may closely mimic septic arthritis, and the two may coexist. Definitive diagnosis requires arthrocentesis: synovial fluid analysis, including cell count with differential, crystal analysis, Gram stain, and culture. Septic arthritis typically produces a white blood cell count greater than 50,000 cells/μL with greater than 75% polymorphonuclear cells, though lower counts do not reliably exclude infection, particularly in early or partially treated cases. Synovial lactate and glucose levels add diagnostic value in equivocal cases. ED management includes arthrocentesis (diagnostic and therapeutic), broad-spectrum antibiotic administration after aspiration (typically covering *Staphylococcus aureus*), emergent orthopedic consultation for surgical washout consideration, and hospital admission. Blood cultures should be obtained before antibiotic administration. Delays in antibiotic therapy and joint decompression are associated with articular cartilage destruction and permanent joint damage [14].

Compartment Syndrome

Acute compartment syndrome (ACS) occurs when intracompartmental pressure within a closed fascial space exceeds the perfusion pressure of the contained tissues, resulting in ischemia, necrosis, and irreversible damage to muscle and nerve. It is a true surgical emergency. The leg is the most commonly affected site (the anterior compartment most frequently), followed by the forearm, thigh, and foot. The classic early symptom is severe pain, disproportionate to the apparent injury, and progressive. Pain with passive stretch of the muscles within the affected compartment is the most sensitive early clinical sign and should be actively tested in any patient at risk [15]. A tense, woody-feeling compartment on palpation is a reliable finding when present. Paresthesias and weakness develop as the ischemia progresses. The classic 5 P's (pain, paresthesias, pressure, pallor, pulselessness) describe the full constellation, but pulselessness is a late and ominous finding that should never be awaited before acting.

The diagnosis is primarily clinical, but compartment pressure measurement is a valuable adjunct when the clinical picture is unclear or the patient is obtunded. Normal compartment pressure is less than 10 millimeters of mercury (mmHg). Fasciotomy is indicated when the absolute compartment pressure exceeds 30 mmHg or when the difference between diastolic blood pressure and compartment pressure (the delta pressure) is less than or equal to 30 mmHg [16]. The delta pressure threshold is particularly important in hypotensive patients, in whom a compartment pressure of 20 mmHg may produce ACS.

Emergency management begins with immediate removal of all constrictive dressings, casts, and circumferential bandages and elevation of the limb to the level of the heart (not above, as this reduces perfusion). Supplemental oxygen should be applied. Orthopedic surgery should be notified immediately, and transfer to the operating room for emergent fasciotomy should occur without delay. The window for reversal of ischemic injury is narrow: permanent muscle and nerve damage may occur within six to eight hours of onset [16].

Posterior Shoulder Dislocation

Posterior shoulder dislocation accounts for only 2-4% of all glenohumeral dislocations but is missed in approximately 50-79% of cases at initial presentation, making it one of the most commonly missed orthopedic diagnoses in the ED [16]. The mechanism typically involves forceful internal rotation and adduction of the shoulder, most classically associated with seizure, electrocution, and fall on an outstretched internally rotated arm. The classic presentation is a patient holding the arm in internal rotation and adduction with inability to externally rotate. 

The diagnosis is missed primarily because standard AP shoulder radiographs may appear deceptively normal or show only subtle abnormalities, including the "trough sign" (impaction fracture of the humeral head) and the "rim sign" (widened glenohumeral joint space). The critical diagnostic step is obtaining a true axillary view or Y-scapular view, on which the posterior position of the humeral head relative to the glenoid is readily apparent. Every emergency physician should be aware that a patient with the arm locked in internal rotation after a seizure or electrocution has a posterior dislocation until proven otherwise. CT is diagnostic when plain film views are inadequate or the diagnosis remains uncertain [16].

Special populations

Pediatric Patients

MSK injury in children differs fundamentally from adult injury because of the presence of the physis (growth plate), which is the weakest point in the immature skeleton. Physeal cartilage has lower tensile strength than the adjacent ligamentous structures, meaning that forces that would produce ligamentous sprains in adults instead cause physeal fractures in children and adolescents. Clinically, apparent sprains in skeletally immature patients should prompt careful evaluation for physeal injury. The Salter-Harris classification describes physeal fractures by the relationship of the fracture line to the growth plate. Type I (transverse physeal fracture, normal radiograph) and type II (fracture through the physis exiting through the metaphysis) are the most common and generally carry a good prognosis. Types III-V involve the epiphysis or crush the physis and carry a higher risk of growth disturbance. Tenderness directly over the physis with a normal radiograph should be treated as a Salter-Harris type I fracture in children [6].

Specific patterns to recognize in pediatric patients include the following: lateral ankle physeal fractures presenting as apparent ankle sprain (tenderness at the distal fibular physis, not the anterior talo-fibular ligament); toddler's fracture (nondisplaced spiral tibial fracture in young children with refusal to walk); lateral condyle fracture of the humerus (may appear subtle and has high risk of displacement and nonunion if not recognized); and supracondylar humerus fractures, the most common pediatric elbow fracture, which carry significant risk of anterior interosseous nerve injury and brachial artery injury. Careful anterior interosseous nerve testing (flexor pollicis longus and flexor digitorum profundus to the index finger, making an "OK" sign) and pulse assessment are mandatory in children presenting with supracondylar fractures. Finally, non-accidental trauma (NAT) should be considered in any child with an injury pattern inconsistent with the developmental stage or the reported mechanism, multiple fractures at different stages of healing, metaphyseal corner fractures, posterior rib fractures, or fractures in non-ambulatory children. When NAT is suspected, a skeletal survey should be obtained, and appropriate child protective services involvement initiated [6].

Elderly Patients

In elderly patients, reduced bone mineral density (osteoporosis), impaired proprioception, diminished muscle mass, polypharmacy, and comorbid medical conditions significantly alter both the pattern and severity of MSK injury. Low-energy mechanisms that would produce minor injuries in younger adults can cause significant fractures in elderly patients, and the threshold for imaging should be lower in this population. Hip fracture is among the most consequential MSK diagnoses in the elderly, carrying a one-year mortality rate of approximately 20-30% and significant rates of permanent functional decline among survivors [17]. The injury typically follows a fall from standing height or less. Patients present with hip or groin pain, inability to bear weight, and external rotation and shortening of the affected leg in displaced fractures. However, nondisplaced femoral neck fractures may present with only groin or anterior thigh pain and preserved ability to bear weight with a limp, making the clinical diagnosis challenging. As discussed in the imaging section, a normal AP pelvis radiograph does not exclude occult hip fracture in an elderly patient with inability to bear weight or significant groin pain, and MRI (or CT as an alternative with lower sensitivity) should be pursued before discharge [17].

Distal radius, vertebral compression, and proximal humerus fractures are also common osteoporotic injuries after low-energy trauma. In elderly patients, clinicians should consider a pathologic fracture when pain is disproportionate, trauma is minimal, or systemic symptoms or a history of malignancy is present. Fall risk and baseline functional status should also be incorporated into ED disposition planning [17].

ED management principles

Once an MSK injury has been identified or strongly suspected, emergency management should address four practical priorities: analgesia, structural protection of the injured structure, identification of injuries requiring immediate intervention, and clear disposition with structured follow-up. These priorities apply regardless of the specific injury and should guide the management plan from the time of diagnosis through discharge.

Analgesia

Adequate analgesia is both a patient-centered priority and a diagnostic necessity, as it allows for more reliable repeat examination. Delayed analgesia for MSK injuries is a recognized quality problem in emergency care [18]. For mild to moderate injuries, acetaminophen and non-steroidal anti-inflammatory drugs (NSAIDs) represent the safest first-line options and are effective for most acute MSK pain when administered at adequate doses. NSAID use should be individualized according to injury type, pain intensity, comorbidities, renal, gastrointestinal, or cardiovascular risk, and functional goals.

More severe pain, fractures, and injuries requiring procedural intervention may require short-acting opioid analgesia, regional nerve blocks, or procedural sedation. Hematoma blocks (injection of local anesthetic directly into the fracture hematoma) are safe, effective, and underutilized for distal radius and ankle fractures. Femoral nerve blocks substantially reduce pain and opioid requirements for proximal femur and femoral shaft fractures. Regional nerve block techniques are increasingly within the scope of emergency medicine practice and can provide superior analgesia with fewer systemic side effects than parenteral opioids.

Non-pharmacologic measures, including ice, elevation, and temporary offloading, remain valuable adjuncts, particularly in the first 24-48 hours after injury, where they reduce swelling and pain. Current evidence favors the PEACE & LOVE (Protect, Elevate, Avoid anti-inflammatory modalities, Compress, Educate and Load, Optimism, Vascularisation, and Exercise) framework over the older RICE (Rest, Ice, Compression, and Elevation) model, emphasizing protection, elevation, avoidance of anti-inflammatory modalities in the very early phase, compression, education, optimal loading, vascularization, and exercise in subsequent management [19]. However, the utilization of the PEACE & LOVE framework vs the use of RICE therapy should be dependent on injury type, recovery phase, and clinical context.

Immobilization and Reduction

Immobilization should be tailored to the suspected injury rather than defaulting to a generic splint. Fractures and unstable ligamentous injuries require rigid immobilization with a plaster or fiberglass splint, while complete circumferential casting in the acute phase is generally avoided because progressive swelling may cause compartment syndrome. Tendon ruptures require positioning that reduces tension on the injured tendon: plantarflexion for the Achilles, extension for the patellar tendon, and thumb spica for the scaphoid. Minor soft tissue injuries may be managed with functional bracing or a compression wrap when fracture has been excluded, and stability is preserved.

Reduction should be performed promptly for dislocations and significantly displaced fractures, particularly when there is skin tenting, neurovascular compromise, or severe pain. Pre-reduction neurovascular assessment should be documented, procedural sedation or regional anesthesia should be used as appropriate, and post-reduction radiographs should be obtained to confirm reduction and identify associated fractures. Persistent neurovascular deficit after reduction, irreducible dislocation, and unstable post-reduction alignment require urgent specialist involvement from the ED.

Weight-Bearing Instructions and Ambulation

Clear, specific weight-bearing instructions are an essential component of safe discharge. Vague instructions to "take it easy" are insufficient and contribute to patient non-compliance and secondary injury. Instructions should specify one of four categories: NWB, toe-touch weight-bearing, partial weight-bearing, or weight-bearing as tolerated. If the patient is prescribed NWB status with crutches, hands-on crutch training should be provided in the ED and confirmed before discharge. Patients who cannot safely use crutches due to upper extremity injury, poor balance, or cognitive impairment may require alternative mobility aids or inpatient admission.

Reassessment After Intervention

A structured reassessment should be documented after analgesia, reduction, splint placement, or prolonged observation. Repeat neurovascular examination is mandatory after any manipulation or reduction. The adequacy of pain control, the fit and alignment of the splint, and the post-reduction neurovascular status should all be explicitly documented. For high-risk injuries such as displaced fractures and dislocations, reassessment should occur within 30-60 minutes of intervention. Persistent pain escalation in a splinted extremity should prompt evaluation for compartment syndrome.

Disposition and follow-up

Safe disposition requires integration of multiple factors: the severity and stability of the injury, adequacy of pain control, patient functional status and home support, risk of delayed complications, and the urgency of subspecialty follow-up. A practical triaging framework divides patients into those requiring immediate specialist involvement or admission, those requiring urgent outpatient follow-up, and those appropriate for routine follow-up.

Indications for Hospital Admission or Immediate Consultation

Immediate orthopedic consultation or hospital admission is generally indicated for: open fractures; compartment syndrome; septic arthritis; dislocations with persistent neurovascular compromise or failed closed reduction; unstable fractures requiring surgical stabilization; significant skin tenting over a fracture; suspected vascular injury requiring urgent vascular surgery evaluation; and patients who cannot safely comply with NWB or immobilization requirements at home. Patients with ACS must be taken emergently to the operating room; delays should be avoided once the diagnosis is established.

Urgent Outpatient Follow-Up (48-72 Hours)

Urgent follow-up is appropriate for injuries where early reassessment may change management, even if the patient is clinically stable for discharge. This category includes suspected Achilles tendon rupture (to confirm diagnosis and initiate definitive management); suspected Lisfranc injury (to obtain weight-bearing films if not done in the ED and arrange specialist evaluation); possible scaphoid fracture with negative initial radiographs (for repeat imaging or advanced imaging); tibial plateau fractures managed without admission; and patients with reduced dislocations where alignment, swelling, or neurovascular status should be re-examined within days.

Routine Follow-Up (One to Two Weeks)

Routine follow-up is appropriate for stable fractures managed conservatively, uncomplicated ligament sprains, and overuse-related presentations when fracture, instability, and neurovascular compromise have been reasonably excluded. These patients still require structured discharge instructions and return precautions, but do not require rapid specialty evaluation. Exceptions should be made for injuries in elderly patients or those with medical comorbidities that may complicate healing or compliance.

Discharge Counseling and Return Precautions

Discharge counseling is a core component of safe MSK disposition. Patients should receive explicit information on: the specific diagnosis or working diagnosis; the level of activity and weight-bearing permitted; how and when to use ice and elevation; medication instructions, including name, dose, frequency, and expected duration of use; and the follow-up plan, including who to contact and when.

Return precautions should be specific and concrete. Patients should be instructed to return for: increasing pain disproportionate to the injury trajectory; new or worsening numbness, tingling, or weakness distal to the injury; skin color change (pallor, cyanosis, or mottling); tightening of the splint or cast; drainage, odor, or signs of wound infection; fever greater than 38°C (100.4°F); or inability to bear weight when they were previously permitted to do so. Patients with suspected occult fractures should be explicitly counseled that their initial radiograph may not demonstrate the injury and that repeat imaging is planned at follow-up.

Common cognitive errors and diagnostic pitfalls

Diagnostic errors in MSK injury evaluation often result from premature closure, anchoring bias, or overreliance on imaging rather than from lack of knowledge alone. In the ED, time pressure, high patient volume, and initially reassuring examinations can contribute to missed injuries.

One of the most common pitfalls is anchoring on a seemingly benign diagnosis such as "ankle sprain" without reassessing for occult fracture, syndesmotic injury, Lisfranc injury, or tendon rupture. Patients with Achilles tendon rupture, for example, may retain partial plantarflexion and still ambulate, leading clinicians to underestimate the severity of injury if a Thompson test is not specifically performed. Another common error is overreliance on normal plain radiographs. Occult fractures of the scaphoid, tibial plateau, hip, and midfoot may not be visible on initial imaging. Missed fractures remain among the most common diagnostic imaging errors in emergency medicine, particularly in anatomically complex regions, such as the wrist, foot, ankle, and hip [20]. Persistent inability to bear weight, disproportionate pain, significant swelling, or high-risk mechanisms should prompt reconsideration of the diagnosis even when radiographs appear normal.

Finally, emergency physicians should avoid evaluating MSK injuries in isolation from the overall clinical picture. Neurovascular compromise, compartment syndrome, septic arthritis, and open injuries may initially present subtly but require urgent recognition and intervention. Maintaining a structured and systematic approach helps reduce diagnostic error and improve patient safety.

## Conclusions

This review is intended as a practical narrative overview of acute MSK injury evaluation in the ED. As a narrative review, it is limited by selective inclusion of the literature and is not intended to represent a comprehensive systematic review. The principles discussed should be integrated with clinical judgment and local practice patterns. Acute MSK injuries represent one of the largest and most heterogeneous categories of ED presentations, encompassing a spectrum of pathology from minor contusions to immediately life- or limb-threatening emergencies. Providing consistently high-quality care across this spectrum requires a systematic diagnostic approach applicable across multiple anatomic regions and injury types, rather than a piecemeal memorization of individual diagnoses. The framework presented in this review integrates five core domains: mechanism of injury analysis, functional assessment, structured regional physical examination, targeted imaging guided by validated clinical decision rules, and evidence-based management with deliberate disposition planning. Applying this framework consistently allows emergency physicians to efficiently identify clinically important injuries, avoid unnecessary imaging, and reduce the risk of missed diagnoses.

Several high-risk injuries deserve particular vigilance: Achilles tendon rupture frequently masquerades as ankle sprain; scaphoid fractures are occult on initial radiographs in 10-20% of cases; tibial plateau fractures may be subtle on plain film and carry significant associated injuries; Lisfranc injury is the most commonly missed foot injury; septic arthritis demands prompt recognition and joint aspiration; and compartment syndrome requires immediate surgical intervention once recognized. Posterior shoulder dislocation is a diagnosis that emergency physicians must actively seek, particularly after a seizure or electrocution. Special populations - pediatric patients with physeal injuries and elderly patients with osteoporosis-related fractures - require modified clinical approaches that reflect the unique biomechanical and physiologic characteristics of these groups. Emergency physicians must maintain awareness of non-accidental trauma in children and must apply a lower threshold for advanced imaging in the elderly patient, where there is a normal plain radiograph but a clinically convincing presentation. Well-communicated discharge planning, including specific weight-bearing instructions, structured return precautions, and explicit follow-up arrangements, is as important as the diagnostic evaluation itself. The emergency physician's role extends from recognition and initial management through safe disposition, and the quality of the handoff to the outpatient or inpatient setting determines the patient's ultimate outcome as much as any specific intervention performed in the department.
